# Left atrial emptying fraction predicts recurrence of atrial fibrillation after radiofrequency catheter ablation

**DOI:** 10.1371/journal.pone.0191196

**Published:** 2018-01-24

**Authors:** Chung-Chuan Chou, Hui-Ling Lee, Po-Cheng Chang, Hung-Ta Wo, Ming-Shien Wen, San-Jou Yeh, Fen-Chiung Lin, Yi-Ting Hwang

**Affiliations:** 1 Division of Cardiology, Department of Internal Medicine, Chang Gung Memorial Hospital, Linkou, Taiwan; 2 Chang Gung University College of Medicine, Taoyuan, Taiwan; 3 Department of Anesthesia, Chang Gung Memorial Hospital, Taipei, Taiwan; 4 Department of Statistics, National Taipei University, Taipei, Taiwan; Harvard Medical School, UNITED STATES

## Abstract

**Background:**

Compared with left atrial (LA) dimension, LA emptying fraction (LAEF) has received less emphasis as a predictor of atrial fibrillation (AF) recurrence after radiofrequency catheter ablation (RFCA). In addition, patients experiencing post-RFCA AF recurrence may respond to previously ineffective antiarrhythmic drugs (AADs). Classifying these patients into a third RFCA outcome category is recommended.

**Objective:**

To identify predictors of RFCA outcome classified into three categories, and to build proportional odds logistic regression models for clinical applicability to predict AF recurrence.

**Methods:**

Data were retrospectively collected from 483 consecutive patients with drug-refractory AF undergoing RFCA (328 men; age 58.4 ± 11.5 years; 383 paroxysmal). Patients were classified into 3 groups based on the last RFCA outcome: group 1, free from AF without AADs; group 2, free from AF with AADs; and group 3, recurrence of AADs-refractory atrial tachyarrhythmia.

**Results:**

After a mean follow-up duration of 64.5 ± 43.2 months and mean ablation procedure number of 1.37 ± 0.68, the RFCA outcome showed 76.0%, 9.5% and 14.5% of patients in groups 1, 2, and 3, respectively. In multivariate analysis, LAEF was the most stable and important predictor of AF recurrence, followed by body mass index, stroke, AF duration, mitral regurgitation, and LA linear ablation. For patients undergoing repeat RFCA, LAEF was the only independent predictor (cutoffs: 43% and 35% for groups 1 and 3, respectively).

**Conclusion:**

LAEF provides optimal prognostic information regarding the risk stratification of AF patients undergoing RFCA.

## Introduction

Enlarged left atrium (LA) measured by echocardiography may predict the recurrence of atrial fibrillation (AF) after radiofrequency catheter ablation (RFCA) [1, 2]. However, the role of LA dimension in predicting the success of AF ablation remains controversial; thus, recent recommendations no longer include LA size as a criterion for the selection of AF patients as candidates for RFCA [3]. Despite being recognized as a predictor of AF recurrence [4, 5], less emphasis has been given to LA emptying fraction (LAEF). Our preliminary clinical experiences showed that some patients with a dilated LA but preserved LAEF were free from AF, and some other patients with a normal-sized LA but reduced LAEF experienced AF recurrence after RFCA. In addition, previous studies have identified predictors of AF recurrence by using multivariate analyses without taking antiarrhythmic drugs (AADs) treatment into considerations [6–8]. However, patients experiencing post-RFCA AF recurrence may respond to previously ineffective AADs. It is recommended that these patients should be classified into a third category of RFCA outcome rather than a binary outcome of “recurrence” or “no recurrence”. Therefore, the purposes of the present study were 1) to identify the clinical factors and echocardiographic parameters that were potential predictors of RFCA outcome, which was classified into 3 categories; and 2) to build proportional odds logistic regression models for clinical applicability to predict AF recurrence.

## Methods

### Study population

In this study, we retrospectively evaluated 483 consecutive patients who underwent RFCA for symptomatic AF refractory to AADs between July 2004 and December 2016 at our institution. Patients who had AF episodes that self-terminated within 7 days were classified as having paroxysmal AF, and those whose AF episodes lasted longer than 7 days were classified as having non-paroxysmal AF. For all patients, detailed medical history regarding AF and related cardiovascular and systemic conditions were obtained. On the basis of RFCA outcome, we divided patients into 3 groups: group 1, no AF recurrence; group 2, recurrence of atrial tachyarrhythmia responsive to AADs; and group 3, recurrence of atrial tachyarrhythmia refractory to AADs. The potential predictors of AF recurrence were identified from clinical and echocardiographic data. Body mass index (BMI) was categorized into 3 degrees as follows: < 25 kg/m^2^ (BMI degree (BMId) = 1), 25 kg/m^2^ to 30 kg/m^2^ (BMId = 2), and ≥ 30 kg/m^2^ (BMId = 3). Patients in whom AF could not be converted into sinus rhythm after RFCA, who had severe valvular disease requiring surgery, or who received surgical MAZE previously were excluded from this study. The Institutional Review Board of Chang Gung Memorial Hospital approved the study protocol, and written informed consent was obtained from all patients.

### Electrophysiological study and RFCA

All patients received RFCA under general anesthesia. RFCA was performed using a 3D electroanatomical mapping system (CARTO, Biosense-Webster, Diamond Bar, CA, USA) to support the creation and validation of ablation lesions. A 3.5-mm open-tip irrigated CARTO catheter and a circular catheter (Lasso, Biosense Webster) were percutaneously introduced through the right femoral vein. For paroxysmal AF, we analyzed the initiating foci of AF by identifying spontaneous ectopic beats through isoproterenol infusion (1–4 μg/min). Radiofrequency energy was continuously delivered to circumferentially encircle the ipsilateral superior and inferior pulmonary veins (PVs), and the end point was the elimination or dissociation of all PV potentials. When non-PV foci were identified, ablation of the earliest sites was performed. Additional LA linear ablation (LA_abl_) was performed at the discretion of the operator after PV isolation. This often consisted of ablation lines to create conduction block across the LA roof and along the region between the lateral mitral annulus and left inferior PV. External cardioversion was performed to restore sinus rhythm if RFCA failed to convert AF.

## Echocardiography

On the next day after RFCA, 2D echocardiographic images were obtained in sinus rhythm, which were used as the baseline echocardiographic data for predicting the overall success of RFCA. Serial echocardiographic examinations were performed at 1, 3, 6, and12 months and then every 6 months after RFCA. These examinations were performed using a commercially available ultrasound scanner (Vivid 7 or 9, General Electric Medical Health, Waukesha, WI, USA) with a 2.5-MHz phased-array transducer. LA and left ventricle (LV) measurements were obtained according to the guidelines of the American Society of Echocardiography.[9] The 2D LA volume was measured from the apical 4-chamber view [10]. The LA maximum volume (V_max_) was defined as the largest volume just before mitral valve opening and the LA minimum volume (V_min_) was defined as the smallest possible volume in ventricular diastole. LAEF was calculated using the following formula: (V_max_-V_min_)/V_max_× 100 [11, 12]. Mitral regurgitation (MR) was categorized into 2 grades (grade 0: ≤ mild; grade 1: ≥ mild to moderate).

### Follow-up and definition of recurrence

Follow-up was conducted at 1 week, 1 month, 3 months, 6 months, and 12 months and then every 3–6 months after RFCA and whenever required because of the symptoms of AF. Serial 12-lead electrocardiograms and 24-hour Holter ambulatory electrocardiogram were recorded after RFCA and when patients exhibited symptoms of palpitation. Recurrence was defined if patients experienced self-reported typical palpitation episodes (> 30 s) or atrial tachyarrhythmia on a 12-lead electrocardiogram, Holter monitoring, or pacemaker/implantable cardioverter-defibrillator interrogation (where available) at the follow-up visit. AADs were prescribed to patients with recurrent atrial tachyarrhythmia. Repeat RFCA was advocated in patients who remained symptomatic despite use of AADs. The primary outcome of the present study was the recurrence of AF or procedure-related atrial tachycardia after the last RFCA.

### Statistical analysis

Continuous variables were summarized in term of mean ± standard deviation and categorical variables were represented by numbers and percentages. To assess the association between variables and the status of RFCA outcome, we performed analysis of variance procedures for continuous variables and the chi-square test or likelihood ratio test for categorical variables. Variables with *P* < 0.05 in bivariate analysis were considered in multivariate regression models. Proportional odds logistic regression models were built and a stepwise method (selection for entry criteria = 0.05; selection for stay criteria = 0.1) was used to identify independent predictors. The odds ratio and 95% confidence interval (CI) were calculated for each variable. The C statistic was used to evaluate the model’s discriminatory ability in predicting AF ablation outcome. Split-sample cumulative cross-validation (repeat for 500 times) was used to test the reproducibility of model fitting. The percentage of significance of independent predictors, average C statistics, correct specified percentage, and results of correct and incorrect percentage for the 3 categories were calculated to evaluate the reproducibility of the model. Since LAEF was likely highly correlated to LA volume, we created a single best multivariate regression model without including any LA measurement as the nested model first. Then we added LAEF, V_max_ and V_min_ in the nested model separately to create three new models. Comparisons of these three models to the nested model were made by measurements of AIC (Akaike information criterion), -2 Log L (-2 Log likelihood value), and SC (Bayesian Schwarz information criterion) as well as the C statistic. Statistical analyses were performed using SAS version 9.4 (SAS institute Inc., Cary, NC, USA).

## Results

### RFCA outcome

Our study population comprised 483 consecutive AF patients (mean age 58.4 ± 11.5 years, mean duration of AF before RFCA 3.8 ± 3.3 years, 67.9% men, and 79.3% paroxysmal AF). The mean follow-up duration was 64.5 ± 43.2 months (median: 55 months). A total of 662 procedures were performed, and 103 (21.3%), 26 (5.4%), 4 (0.8%) and 3 (0.6%) patients received RFCA 2, 3, 4 and 5 times, respectively. Detailed information of RFCA time in three groups was summarized in Table 1. There was no significant difference of the distribution of ablation time among three groups (*P* = 0.202). LA_abl_ was performed in 209 (43.3%) patients. These patients had a higher percentage of non-paroxysmal AF than those who did not undergo LA_abl_ (40.2% vs. 5.8%, *P*<0.001). Moreover, a larger LA dimension (LAD) (43.6 ± 7.0 vs. 40.1 ± 6.0 mm, *P*<0.001), V_max_ (69.1 ± 28.2 vs. 57.1 ± 21.8 ml, *P*<0.001), V_min_ (38.4 ± 24.7 vs. 26.1 ± 15.5 ml, *P*<0.001) and poorer LAEF (47.9% ± 13.5% vs. 56.3% ± 10.5%, *P*<0.001) were noted in patients who underwent LA_abl_ than in those who did not undergo LA_abl_. After the last RFCA, freedom from AF was achieved in 413 (85.5%) patients, comprising 367 (76.0%, group 1) patients who did not require AADs and 46 (9.5%, group 2) patients who required AADs for AF control. Group 3 contained 70 (14.5%) patients, comprising 14 (2.9%) patients with recurrence of atrial tachycardia and 56 (11.6%) patients with AF. The overall procedure-related major complication rate was 2.3%: 10 (2.1%) patients had cardiac tamponade requiring pericardial drainage and 1 (0.2%) patient had a minor stroke.

**Table 1:** Distribution of ablation time among the study groups.

	Group 1 (AF (-), AADs (-))	Group 2 (AF (-), AADs (+))	Group 3 (AF (+))	Total
**RFCA 1 time**	269 (73.3%)	32 (69.6%)	46 (65.7%)	347 (71.8%)
**RFCA 2 times**	76 (20.7%)	10 (21.7%)	17 (24.3%)	103 (21.3%)
**RFCA 3 times**	19 (5.2%)	2 (4.3%)	5 (7.1%)	26 (5.4%)
**RFCA 4 times**	1 (0.3%)	2 (4.3%)	1 (1.4%)	4 (0.8%)
**RFCA 5 times**	2 (0.5%)	0 (0%)	1 (1.4%)	3 (0.6%)

Group 1: no AF recurrence; Group 2: recurrence of atrial tachyarrhythmia responsive to antiarrhythmic drugs; Group 3: recurrence of atrial tachyarrhythmia refractory to antiarrhythmic drugs (AADs). RFCA: radiofrequency catheter ablation.

### Predictors of overall success

Patient characteristics were summarized in Table 2. In bivariate analysis, the female sex, BMI ≥30 kg/m^2^, non-paroxysmal AF, longer AF duration (AFD), LA_abl_, tachycardia-bradycardia syndrome, end stage renal disease, rheumatic heart disease, high CHA2DS2VASc score, stroke, larger LAD, larger V_max_, larger V_min_, lower LAEF and LV ejection fraction, thicker interventricular septum, and MR were significant predictors of a poorer RFCA outcome. The multivariate analysis showed that the most influential variable was LAEF, followed by BMI ≥30 kg/m^2^, stroke, AFD, MR, and LA_abl_ (Model A, Table 3). The variables of LA size were not included during an automated stepwise selection procedure when LAEF was included in the model. To evaluate the predictive power of LA size for RFCA outcome, we performed an additional model selection procedure without LAEF. The strongest predictor was then found to be V_min_, followed by V_max_, BMI ≥30 kg/m^2^, stroke, MR, AFD, and tachycardia-bradycardia syndrome (Model B, Table 4). That is, LA size was also a strong predictor of AF recurrence in patients undergoing RFCA. However, the predictive power of Model B was slightly weaker than that of Model A (C statistic: 0.902 vs. 0.913) even if more variables were included in Model B than those in Model A (7 vs. 6). This finding suggested that LAEF was more suitable than LA size for predicting RFCA outcome.

**Table 2:** Clinical and echocardiographic data of the study groups.

	All patients	Group 1 (AF (-), AADs(-))	Group 2 (AF (-), AADs(+))	Group 3 (AF (+))	P
Patient numbers	483	367 (76.0%)	46 (9.5%)	70 (14.5%)	
Age (years)	58.4±11.5	58.1±11.3	61.7±11.4	58.1±12.2	0.125
Gender (males)	328 (67.9%)	262 (79.9%)	22 (6.7%)	44 (13.4%)	0.003
BMI (kg/m^2^)					0.001
<25	232 (48.0%)	184 (50.1%)	19 (41.3%)	29 (41.4%)	
25~30	208 (43.1%)	161 (43.9%)	21 (45.7%)	26 (37.1%)	
≥30	43 (8.9%)	22 (6.0%)	6 (13.0%)	15 (21.4%)	
AF type (paroxysmal)	383 (79.3%)	324 (88.3%)	24 (52.2%)	35 (50.0%)	<0.001
AFD (years)	3.8±3.3	3.6±3.2	3.9±2.8	4.8±3.8	0.035
LA_abl_	209 (43.3%)	140 (38.1%)	31 (67.4%)	38 (54.3%)	<0.001
TBS	56 (11.6%)	33 (9.0%)	6 (13.0%)	17 (24.3%)	0.001
Ablation time	1.37±0.68	1.34±0.64	1.43±0.78	1.49±0.81	0.212
Hypertension	270 (55.9%)	201 (54.8%)	26 (56.5%)	43 (61.4%)	0.587
Diabetes mellitus	72 (14.9%)	54 (14.7%)	6 (13.0%)	12 (17.1%)	0.814
Dyslipidemia	132 (27.3%)	96 (26.2%)	17 (37.0%)	19 (27.1%)	0.301
CAD	26 (5.4%)	20 (5.4%)	2 (4.3%)	4 (5.7%)	0.941
ESRD	24 (5.0%)	12 (3.3%)	4 (8.7%)	8 (11.4%)	0.016
RHD	8 (1.7%)	0 (0%)	3 (6.5%)	5 (7.1%)	<0.001
CHA2DS2VASc	1.66±1.31	1.50±1.23	2.04±1.17	2.21±1.61	<0.001
Stroke	38 (7.9%)	18 (4.9%)	4 (8.7%)	16 (22.9%)	<0.001
Smoking	21 (4.3%)	13 (3.5%)	3 (6.5%)	5 (7.1%)	0.337
COPD	21 (4.3%)	15 (4.1%)	2 (4.3%)	4 (5.7%)	0.840
Echo data					
LAD (mm)	41.6±6.7	40.1±5.8	44.6±6.8	47.4±7.2	<0.001
V_max_ (ml)	62.3±25.5	56.2±18.9	75.2±28.1	85.6±35.8	<0.001
V_min_ (ml)	31.4±20.9	24.8±12.8	43.1±22.3	58.1±28.5	<0.001
LAEF (%)	52.7±12.6	57.3±8.8	44.5±10.9	33.6±9.7	<0.001
IVS (mm)	12.1±2.4	11.9±2.2	12.0±2.4	12.8±3.3	0.028
LVEF (%)	66.5±6.8	67.2±5.7	65.4±5.6	63.8±11.1	<0.001
MR grade					<0.001
≤ Mild	375 (77.6%)	311 (84.7%)	24 (52.2%)	40 (57.1%)	
≥ Mild to moderate	108 (22.4%)	56 (15.3%)	22 (47.8%)	30 (42.9%)	

AADs: antiarrhythmic drugs; AF: atrial fibrillation; Group 1: no AF recurrence; Group 2: recurrence of atrial tachyarrhythmia responsive to antiarrhythmic drugs; Group 3: recurrence of atrial tachyarrhythmia refractory to antiarrhythmic drugs. BMI: body mass index; AFD: AF duration; LA_abl_: left atrial linear ablation; CAD: coronary artery disease; ESRD: end stage renal disease; COPD: chronic obstructive pulmonary disease; LAD: left atrial dimension; V_max_: maximal left atrial volume; V_min_: minimal left atrial volume; LAEF: left atrial emptying fraction; IVS: interventricular septum; LVEF: left ventricular ejection fraction; MR: mitral regurgitation; RHD: rheumatic heart disease; TBS: tachycardia-bradycardia syndrome.

**Table 3:** Multivariate analyses on association of predicting variables and RFCA outcome_Model A (with LAEF).

Analysis of Maximum Likelihood Estimates					
Variables	Estimate	Wald	P value	OR	95% CI of OR
**Intercept (*j* = 3)**	5.6496	50.6767	<0.0001		
**Intercept (*j* = 2)**	7.0643	70.5932	<0.0001		
**LAEF**	-0.1806	126.6595	<0.0001	0.835	0.809~0.861
**BMId**		14.3809	0.0008		
**BMId = 3**	1.7116	13.6676	0.0002	5.538	2.235~13.721
**BMId = 2**	0.1301	0.1849	0.6672	1.139	0.629~2.061
**Stroke**	1.0098	5.7715	0.0163	2.745	1.204~6.256
**AFD**	0.0949	5.1937	0.0227	1.100	1.013~1.193
**MR**	0.6565	4.9086	1.928	1.928	1.079~3.446
**LA**_**abl**_	-0.6326	4.3576	0.0368	0.531	0.293~0.962

**Proportional odds regression model:** log [Pr(*Y* ≥ *j*)/1−Pr(*Y* ≥ *j*)] = *αj* [*αj* = 5.6496, if *j* = 3 (group 3); *αj* = 7.0643, if *j* = 2 (group 2)] − 0.1806*LAEF + 0.0949*AFD + 1.7116 [BMId = 3] + 1.0098 [with stroke] + 0.6565 [MR grade ≥ mild to moderate]– 0.6326 [with LA_abl_], where *j* = 1 (group 1) is the reference group。

**Table 4:** Multivariate analyses on association of predicting variables and RFCA outcome_Model B (without LAEF).

Analysis of Maximum Likelihood Estimates					
Variables	Estimate	Wald	P value	OR	95% CI of OR
**Intercept (*j* = 3)**	-2.9332	31.9254	<0.0001		
**Intercept (*j* = 2)**	-1.6538	11.1142	0.0009		
**V**_**min**_	0.2093	82.7214	<0.0001	1.233	1.178~1.290
**V**_**max**_	-0.1190	46.4044	<0.0001	0.888	0.858~0.919
**BMId**		15.0429	0.0005		
**BMId = 3**	1.7298	14.0737	0.0002	5.639	2.284~13.922
**BMId = 2**	0.1267	0.1819	0.6698	1.135	0.634~2.032
**Stroke**	1.1159	7.1728	0.0074	3.052	1.349~6.9.7
**MR**	0.7825	6.9288	0.0085	2.187	1.221~3.916
**AFD**	0.0978	6.0049	0.0143	1.103	1.020~1.192
**TBS**	0.9006	5.8789	0.0153	2.461	1.188~5.097

AFD: AF duration; BMId: body mass index degree, the BMId = 1 as the reference; CI: confidence interval; LA_abl_: left atrial linear ablation; LAD: left atrial dimension; LAEF: left atrial emptying function; MR: mitral regurgitation; OR: odds ratio; TBS: tachycardia-bradycardia syndrome; V_max_: LA maximum volume; V_min_: LA minimum volume.

**Fig 1:**
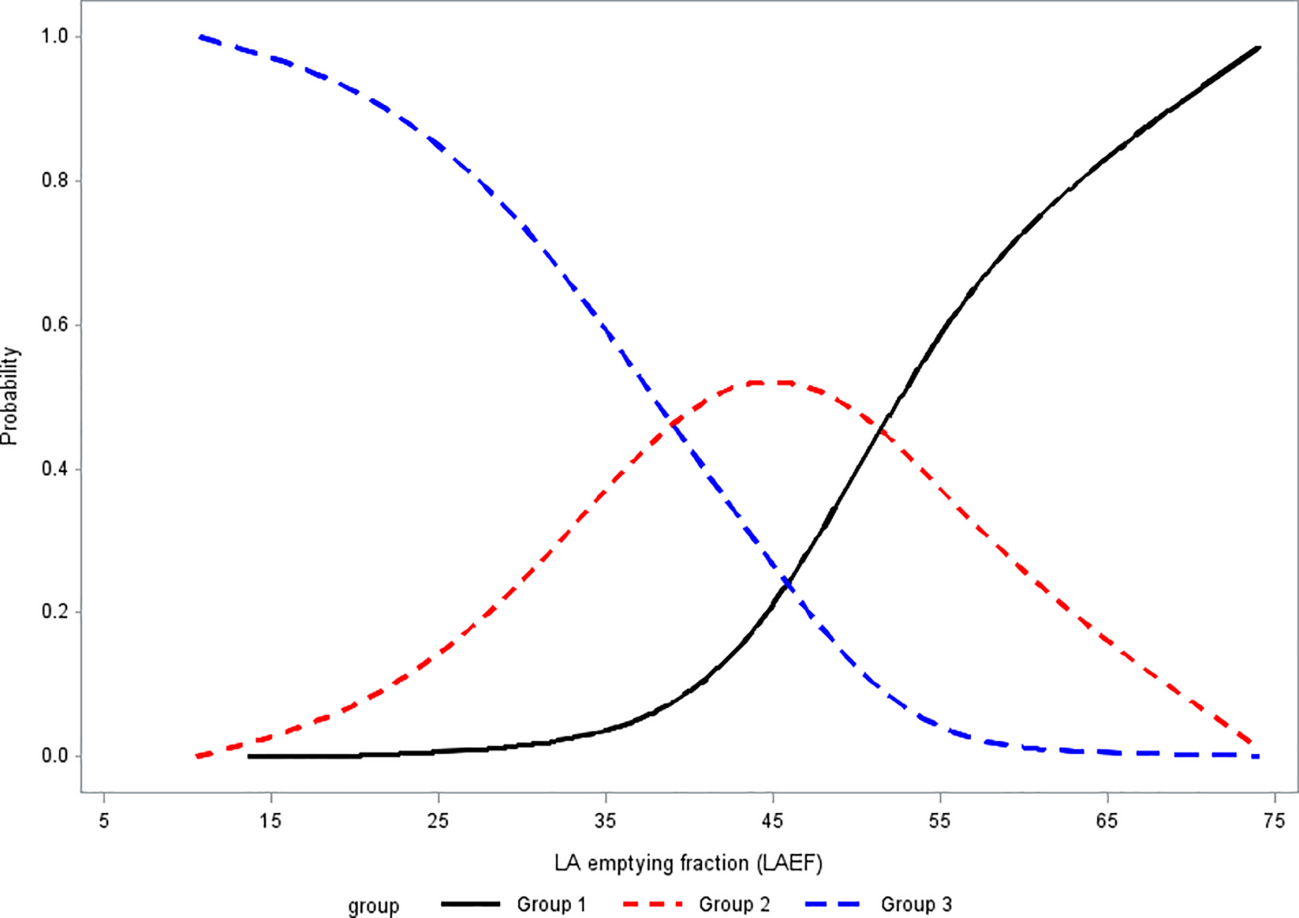
Proportional odds logistic regression on LAEF. The probability of assigning patient to group 1 is high when LAEF is high (black line); the probability of assigning patient to group 3 is high when LAEF is low (blue dashed line); and the peak probability of being group 2 is located at LAEF = 45% (red dashed line).

As shown in Table 3, the odds ratio was less than 1 in LAEF and LA_abl_, that is, a higher LAEF and LA_abl_ decreased the odds of having a severe outcome vs. a non-severe outcome. On the contrary, longer AFD, BMI ≥30 kg/m^2^, stroke and MR grade ≥ mild to moderate increased the odds of having a severe outcome vs. a non-severe outcome. The proportional odds regression model (bottom in Table 3) also provided the cumulative log odds ratio for 1-unit increase in continuous variable predictors. For example, a 1% increase in LAEF predicts a 16.5% decrease in the probability of having a severe RFCA outcome versus a non-severe RFCA outcome. When *j* = 3, log[P(Y ≥3)/P(Y<3)] equals to log[P(Y = 3)/P(Y = 2) + P(Y = 1)]; when *j* = 2, log[P(Y ≥2)/P(Y<2)] equals to log[P(Y = 2) + P(Y = 3)/P(Y = 1)]. In turn, we can obtain the odds of being group 2 by cumulative odds (j = 2) minus cumulative odds (j = 3), and the odds of being group 1 by 1 minus cumulative odds (j = 2) (A clinical use version named “Calculator of RFCA outcome probability” is included in S1 File). Fig 1 shows the fitted probability of assigning patients to each group in terms of LAEF based on the fitted model. When patients have a high LAEF, the probability of assigning patients to group 1 is high; on the contrary, when patients have a low LAEF, the probability of assigning patients to group 3 is high.

The stability of the significance of predictors tested by using the cumulative cross-validation procedure is shown in Table 5. LAEF was the most stable and important predictor and was significant for all 500 random samples, followed by stroke, BMId, AFD, LA_abl_, and MR. The mean C statistic was 0.877 ± 0.014, indicating the overall predictive power and generalizability of the model were excellent. The average of the correct specified percentage was 66.56 ± 2.95%. Table 6 summarizes the average results for correct and incorrect percentage for each category, which showed that the model had a slightly weaker discriminatory ability for identifying group 2.

**Table 5:** The estimation results by cumulative cross-validation (repeat 500 times).

Variables	Category	Estimate	Std. error	% of significance
**Intercept** **LAEF** **Stroke**	3 2	4.769 7.626 -0.154 1.601	1.017 1.192 0.022 0.648	100 100 100 76.0
**BMId**	3	1.296	0.614	61.4
**AFD** **LA**_**abl**_ **MR**	2	-0.025 0.131 -0.605 0.580	0.411 0.062 0.408 0.403	0 58.6 22.8 16.8

AFD: AF duration; BMId: body mass index degree; LA_abl_: left atrial linear ablation; LAEF: LA emptying function; MR: mitral regurgitation; Std. error: standard error; % of significance: percentages of significance of the independent predictors during 500 times of split-sample cumulative cross-validation.

**Table 6:** The average results for correct and incorrect percentage for each category by cumulative cross-validation (repeat 500 times).

Observed	Predicted		
	Group 1 (AF (-), AADs (-))	Group 2 (AF (-), AADs (+))	Group 3 (AF (+))
**Group 1**	78.63%	19.62%	1.76%
**Group 2**	21.96%	53.88%	24.16%
**Group 3**	1.30%	28.76%	69.95%

Group 1: no AF recurrence; Group 2: recurrence of atrial tachyarrhythmia responsive to antiarrhythmic drugs; Group 3: recurrence of atrial tachyarrhythmia refractory to antiarrhythmic drugs (AADs).

To clarify the complicated relationship among LAEF and LA volume parameters, and their separate influences on multivariate logistic regression models, we included all variables with *P<0*.*05* except LAEF, V_max_ and V_min_ in bivariate analysis to create a nested multivariate regression model (Model_nested_), and then added LAEF (Model_LAEF_), V_max_ (Model_Vmax_) and V_min_ (Model_Vmin_) separately to create additional three models. The model fit statistics were shown in Table 7. The nested model had all three criteria values above 540. Controlling the significant predictors, Model_LAEF_ had the smallest values of AIC, SC and -2 Log L, followed by Model_Vmin_ and Model_Vmax_, which was consistent with the C statistic results. All the indices confirmed that Model_LAEF_ was the best model.

**Table 7:** Comparisons of four multivariate logistic regression models in the performance of risk assessment.

Criterion	Model_nested_	Model_LAEF_	Model_Vmax_	Model_Vmin_
**AIC**	576.135	426.391	561.431	517.528
**SC**	643.016	497.451	632.491	588.589
**-2 Log L**	544.135	392.391	527.431	483.528
**C statistic**	0.835	0.920	0.845	0.883

### Predictors of procedure success in patients undergoing repeat RFCA

Table 8 summarized the characteristics of patients undergoing repeat RFCA. We selected the recent echocardiographic images obtained before repeat RFCA for analysis. In bivariate analysis, age, sex, AF type, rheumatic heart disease, LA size, LAEF, and MR were significant predictors of RFCA outcome. The multivariate analysis revealed that LAEF was the only independent predictor of the outcome of repeat RFCA (odds ratio: 0.843, 95% CI: 0.801–0.887, *P*<0.0001). The proportional odds logistic regression model was log[Pr(*Y* ≥ *j*)/1 − Pr(*Y* ≥ *j*)] = *αj* [*αj* = 6.0534, if *j* = 3; *αj* = 7.2899, if *j* = 2; *j*, outcome group] − 0.1712*LAEF. When LAEF ≥ 43%, the model classified patients into group 1; when LAEF ≤ 35%, the model classified patients into group 3. The C statistic of this model was 0.863. The cumulative cross-validation procedure revealed a mean C statistic of 0.799 ± 0.032. This finding indicated that the overall predictive power and generalizability of the model were good. The average of the correct specified percentage was 59.72% ± 5.05%. Table 9 summarizes the average results for correct and incorrect percentage for each category, revealing that the model had a relatively weaker discriminatory ability in identifying group 2 and group 3.

**Table 8:** Clinical and echocardiographic data of AF patients underwent at least two RFCA procedures.

	All patients	Group 1 (AF (-), AADs(-))	Group 2 (AF (-), AADs(+))	Group 3 (AF (+))	P
Patient numbers	136	98 (72.1%)	14 (10.3%)	24 (17.6%)	
Age (years)	57.8±10.6	58.6±9.2	60.6±13.9	52.5±12.5	0.022
Gender (males, %)	90 (66.2%)	69 (76.7%)	5 (5.6%)	16 (17.8%)	0.037
BMI (kg/m2)					0.215
<25	59 (43.4%)	46 (46.9%)	5 (35.7%)	8 (33.3%)	
25~30	64 (47.1%)	46 (46.9%)	7 (50.0%)	11 (45.8%)	
>30	13 (9.6%)	6 (6.1%)	2 (14.3%)	5 (20.8%)	
AF type (paroxysmal)	105 (77.2%)	84 (85.7%)	8 (57.1%)	13 (54.2%)	0.001
AFD (years)	4.3±3.4	4.3±3.5	3.7±2.6	4.9±3.4	0.595
LA_abl_	80 (58.8%)	53 (54.1%)	12 (85.7%)	15 (62.5%)	0.073
TBS	16 (11.8%)	10 (10.2%)	3 (21.4%)	3 (12.5%)	0.523
Ablation times	2.32±0.64	2.28±0.59	2.43±0.76	2.42±0.78	0.496
Hypertension	81 (59.6%)	61 (62.2%)	7 (50.0%)	13 (54.2%)	0.573
Diabetes mellitus	15 (11.0%)	11 (11.2%)	0 (0.0%)	4 (16.7%)	0.138
Dyslipidemia	39 (28.7%)	26 (26.5%)	5 (35.7%)	8 (33.3%)	0.666
CAD	7 (5.1%)	6 (6.1%)	1 (7.1%)	0 (0.0%)	0.244
ESRD	7 (5.1%)	3 (3.1%)	1 (7.1%)	3 (12.5%)	0.217
RHD	3 (2.2%)	0 (0%)	1 (7.1%)	2 (12.5%)	0.020
CHA2DS2VASc	1.56±1.10	1.54±1.05	1.79±1.37	1.50±1.18	0.711
Stroke	10 (7.4%)	7 (7.1%)	0 (0%)	3 (12.5%)	0.231
Smoking	7 (5.1%)	4 (4.1%)	1 (7.1%)	2 (8.3%)	0.680
COPD	9 (6.6%)	8 (8.2%)	0 (0.0%)	1 (4.2%)	0.281
Echo data					
LAD (mm)	41.5±5.9	40.4±5.0	41.9±6.2	45.9±7.0	<0.001
V_max_ (ml)	60.5±24.2	55.3±18.5	61.9±32.0	81.3±29.0	<0.001
V_min_ (ml)	32.3±20.0	26.4±13.6	37.5±24.8	53.3±24.3	<0.001
LAEF (%)	49.8±11.3	54.1±8.0	42.4±11.2	36.4±10.0	<0.001
IVS (mm)	11.9±2.4	11.9±2.4	10.8±1.3	12.7±2.9	0.061
LVEF (%)	65.6±5.6	65.5±5.7	64.4±5.7	66.5±5.2	0.521
MR grade					0.011
≤ Mild	102 (75.0%)	80 (81.6%)	7 (50.0%)	15 (62.5%)	
≥Mild to moderate	34 (25.0%)	18 (18.4%)	7 (50.0%)	9 (37.5%)	

Abbreviations are the same as in Table 2.

**Table 9:** The average results for correct and incorrect percentage for each category by cumulative cross-validation (repeat 500 times) in AF patients underwent at least two RFCA procedures.

Observed	Predicted		
	Group 1 (AF (-), AADs (-))	Group 2 (AF (-), AADs (+))	Group 3 (AF (+))
**Group 1**	69.33%	29.61%	1.06%
**Group 2**	19.22%	53.11%	27.67%
**Group 3**	8.24%	33.60%	58.16%

Group 1: no AF recurrence; Group 2: recurrence of atrial tachyarrhythmia responsive to antiarrhythmic drugs; Group 3: recurrence of atrial tachyarrhythmia refractory to antiarrhythmic drugs (AADs).

## Discussion

The identification of the predictors of maintenance of sinus rhythm after RFCA is highly desirable since it would certainly help cardiac electrophysiologists in reducing unnecessary procedures. The study results reveal that LAEF was the optimal predictor of AF recurrence after RFCA. In patients undergoing repeat RFCA, LAEF was the only independent predictor, with cutoff values of 43% and 35% for group 1 and group 3, respectively. We built proportional odds logistic regression models to distinguish patients without AF recurrence from patients with AADs-responsive or AADs-refractory AF recurrence. Maintenance of AADs treatment would be needed for rhythm control following RFCA if the predicted outcome is classified into group 2. The model also provides more information regarding the effectiveness and indications of repeat RFCA for recurrent AF if patients are concerned of AADs or are unwilling to take these drugs.

### LAEF and LA size for predicting RFCA outcome

Previous studies have reported that LA size is the best predictor of AF recurrence after PV isolation [2, 8]. It implies that a critical mass of atrial tissue plays an important role in the pathophysiological pathway of AF recurrence after RFCA. In the current study, the bivariate analysis revealed a significant association between LA size and RFCA outcome. However, the multivariate analysis showed that LA size was no longer independently predictive when LAEF was included. Several studies have shown that impaired LA function is associated with post-RFCA AF recurrence [4, 5]. As a result of complex interactions among triggers, perpetuators and substrate [13], AF is frequently associated with low voltage areas, fibrosis, and conduction abnormalities in addition to enlargement of atria, which predispose patients to the development and progression of AF [14]. Furthermore, atrial dysfunction may be an earlier indicator of AF-related changes than atrial enlargement [11]. These can explain why patients with normal LA size but reduced LAEF had a higher risk of AF recurrence after RFCA.

LAEF consists of 1) passive function that occurs in early diastole and represents the conduit phase of LA function, and 2) active function that occurs in the late phase and represents the contractile component of LA. In patients with impaired LV relaxation, increased filling pressure not only declines LA passive function but engenders LA stretch and PV dilation, increasing the risk of AF. In response to decreased early filling, LA active function is augmented to maintain LAEF [15]. That is, a preserved LAEF infers that the atrial myocardium is healthy and can compensate for the decreased early filling, and the AF-related remodeling process may still be in the reversible phase despite LA dilation. This may explain why patients with dilated LA but preserved LAEF had a favorable outcome of AF ablation.

### Obesity and AF ablation outcome

Obesity has been reported to be associated with AF development [16, 17]. Progressive obesity can change the atrial size, conduction, histology and expression of profibrotic mediators, thereby perpetuating spontaneous and more persistent AF [18]. Cai et al. reported that overweight/obesity is associated with a poorer prognosis of AF ablation (odds ratio: 4.71) and can thus serve as an independent predictor of AF recurrence [17]. However, Letsas et al. reported that BMI ≥30 kg/m^2^ patients displayed only a trend of higher rate of AF recurrence than BMI <25 kg/m^2^ patients (*P* = 0.258), and obesity was not an independent predictor of AF recurrence after LA catheter ablation [19]. Our data showed that patients with BMI ≥30 kg/m^2^ had a larger LAD (46.9 ± 7.0 vs. 39.1 ± 6.4 mm, *P*<0.001), V_max_ (77.7 ± 28.3 vs. 56.2 ± 21.9 ml, *P*<0.001), V_min_ (42.9 ± 27.5 vs. 27.5 ± 17.9 ml, *P*<0.001), and poorer LAEF (48.7% ± 16.5% vs. 53.6% ± 12.5%, *P* = 0.061) than patients with BMI <25 kg/m^2^, which may contribute to a higher rate of AF recurrence (*P* = 0.0002). Because BMI is linearly associated with short- and long-term increases in AF risk [16], weight control should be an important strategy for preventing AF recurrence after ablation [20].

### Impaired LAEF underlies poorer RFCA outcome in patients with stroke

It was reported that impaired LAEF increases the risk of paroxysmal AF in patients with cryptogenic stroke [21]. Stroke also has been shown to be associated with stasis and diminished LA appendage flow velocity [22], which reflect underlying atrial myopathy that affects RFCA outcome. In the current study, LAEF was lower in patients with stroke (42.3% ± 13.7% vs. 53.6% ± 12.1%, *P*<0.001), which may account, at least in part, for a poorer AF elimination rate (47.4% vs. 78.4%, *P*<0.001) in these patients.

### AFD and AF ablation outcome

In our study, longer AFD before RFCA was associated with a poor RFCA outcome. Since Wijffels et al. proposed the theory of “AF begets AF” [23], extensive studies have investigated the effect of AF burden on the heart. Progressive atrial remodeling as a result of longer AFD can result in a more severe AF burden. Therefore, the residual arrhythmogenic substrate after RFCA may enhance the possibility of AF recurrence [24]. Especially, it has been reported that RFCA may prevent AF recurrence but does not appear to reverse the underlying substrate contributing to AF [25]. It implies that early timing of RFCA for drug-refractory AF is indicated for a better outcome if rhythm control is the goal.

### MR and AF ablation outcome

Getz et al. reported that MR is associated with an increased risk of AF recurrence after ablation, and its impact is mediated by LA size [26]. In the present study, MR grade ≥ mild to moderate was an independent risk factor for AF recurrence, and was associated with a larger LA size and lower LAEF (*P*<0.001 for all comparisons). These results implied that both LA dilation and impaired LA function contribute to MR’s impact on post-RFCA AF recurrence.

### LA_abl_ and AF ablation outcome

The influence of extensive LA ablation on AF ablation outcome is complex. LA_abl_ has been reported to modify the substrate of AF to improve RFCA outcome [27–30]. But RFCA by itself produces scar, which may have detrimental effects on LA function and negatively affect RFCA outcome. Previously, Substrate and Trigger Ablation for Reduction of Atrial Fibrillation (STAR AF) trial [31] showed that PV isolation alone was inferior to PV isolation plus complex fractionated electrograms ablation for high-burden paroxysmal or persistent AF. But recently, STAR AF II [32] reported that additional LA_abl_ plus complex fractionated electrograms ablation did not significantly reduce AF recurrence when compared with PV isolation alone for persistent AF. In the current study, the bivariate analysis showed a poorer RFCA outcome when LA_abl_ was performed, but the multivariate analysis revealed that additional LA_abl_ improved RFCA outcome. The so-called “Simpson’s paradox” implied that significant interactions occurred between LA_abl_ and patients’ baseline conditions. Our data show that patients who received additional LA_abl_ had a higher percentage of non-paroxysmal AF, larger LAD, and poorer LAEF. Whether a more extensive ablation strategy is indicated in AF patients with highly arrhythmogenic atrial substrate needs further investigation.

### Study limitations

It is challenging to synthesize the studies on AF ablation with respect to extreme heterogeneity of patient characteristics, procedural features, follow-up modalities as well as the complex relationships among factors. In a systemic review and meta-analysis paper by Balk et al.,[33] none of the pre-procedural patient characteristics, such as AF type, AF duration, LVEF, LA diameter, gender, age, presence of structural heart disease and presence of hypertension, is able to predict arrhythmia recurrence at a high level of evidence. But a more recent meta-analysis study by D’Ascenzo et al. reported that valvular AF, LA diameter > 50 mm and recurrence within 30 days were most powerful predictors of recurrence after AF ablation, which could help to better tailor the clinical and interventional strategies.[34] The lack of real predictors of AF recurrence could be related more to the quality and the limitations of the existing literature than to a true absence of association.[35] Our study is a single-tertiary center study with a limited sample size. Therefore, additional studies with larger samples are warranted to confirm our findings. In addition, AF recurrence required ambulatory electrocardiogram documentation at specific time points or when patients exhibited with symptoms; therefore, patients with asymptomatic AF between visits may not have been identified. This may have led to an underestimation of the risk of AF recurrence. However, because we only performed AF ablation for patients with AF-related symptoms, the percentage of “incidental” finding of AF recurrence at regular OPD follow-up visit is very rare. There was only one patient presenting with AF recurrence (documented by a routine 12-lead electrocardiogram) but without significant symptoms at the follow-up visit 9 months post ablation.

## Conclusion

Our study demonstrates that the echocardiographic assessment of LAEF provides optimal prognostic information regarding the risk stratification of AF patients undergoing RFCA. This should be taken into account when selecting patients as candidates for AF ablation, especially for repeat RFCA.

## Supporting information

S1 File Calculator of RFCA outcome probability. The probability of RFCA outcome can be obtained by typing patients' data (LAEF, AF duration) and the values of categorical variables (BMI≥ 30 kg/m2, stroke, LA linear RF, MR degree) in colume B ("B19" to "B24"). When *j* = 3, log[P(Y ≥3)/P(Y<3)] equals to log[P(Y = 3)/P(Y = 2) + P(Y = 1)]; when *j* = 2, log[P(Y ≥2)/P(Y<2)] equals to log[P(Y = 2) + P(Y = 3)/P(Y = 1)], and the probabilities will be shown in "D26" and "E26", respectively. In turn, we can obtain the odds of being group 2 by cumulative odds (j = 2) minus cumulative odds (j = 3) (shown in "E29"), and the odds of being group 1 by 1 minus cumulative odds (j = 2)(shown in "F29").
(XLSX)
